# Impact of utilizing solid recovered fuel on the global warming potential of cement production and waste management system: A life cycle assessment approach

**DOI:** 10.1177/0734242X20978277

**Published:** 2020-12-25

**Authors:** Md Musharof Hussain Khan, Jouni Havukainen, Mika Horttanainen

**Affiliations:** Department of Sustainability Science, Lappeenranta-Lahti University of Technology, School of Energy Systems, Finland

**Keywords:** Waste management, alternative fuel, solid recovered fuel, life cycle assessment, cement production, global warming potential, emission reduction

## Abstract

Cement production is responsible for a significant share of global greenhouse gas (GHG) emissions. A potential option to reduce the cement production emissions is to use alternative fuels which can have also an impact on emissions from the waste management sector. This work investigates the change in global warming potential (GWP) of ordinary Portland cement (OPC) production and affected waste management systems when conventional fuels are partially replaced by solid recovered fuel (SRF) made from commercial and industrial waste (C&IW). A life cycle assessment (LCA) was conducted with a functional unit of 1 metric tonne of OPC production and treatment of 194 kg of C&IW. Data from an existing cement plant have been used, where the share of SRF from total fuel energy demand increased from 0% to 53% between 2007 and 2016. Four scenarios were established with varying waste treatment methods and SRF share in the thermal energy mix of cement production. It was found that GHG emissions decreased by 20% from 1036 kg carbon dioxide (CO2), eq. (functional unit)-1 in Scenario 1 to 832 kg CO2, eq. (functional unit)-1 in Scenario 3. Furthermore, it is possible to reach a reduction of 30% to 725 kg CO_2_, eq. (functional unit)-1 in Scenario by increasing the share of SRF to 80%. In conclusion, significant GHG emissions reduction can be achieved by utilizing SRF in cement production. Especially in the middle-income and low-income countries where waste is dumped to the open landfills, emissions could be reduced without huge investments to waste incineration plants.

## Introduction

Cement is one of the most widely used building materials in the world, with global cement production of 4.7 billion metric tonnes (Mts) in 2016 (CEMBUREAU, 2017). The production of cement is an energy-intensive process with an estimated average thermal energy requirement of 3.4 GJ for 1 tonne clinker globally in 2018 (IEA, 2020b). Cement production is also the second largest industrial carbon dioxide (CO_2_) emitter (International Energy Agency, 2018). In 2016, global cement production generated about 2.2 billion Mts of CO_2_, which accounts for about 8% of the global CO_2_ emissions (Andrew, 2018; Rodgers, 2018 ). Of the total greenhouse gas (GHG) emissions generated by the cement industry, approximately 50% of it was emitted by the calcination process, 40% was associated with the fuel combustion in the calcination process, and the remaining 10% was generated from electricity consumption, preparation, and transportation of raw materials and fuels ( Rodgers, 2018). To reduce GHG emissions from cement production, more emphasis should be given on reducing the clinker-to-cement ratio and increasing utilization of alternative fuels (CEMBUREAU, 2013).

The cement industry utilizes solid recovered fuel (SRF) as alternative fuel, which is usually produced from municipal solid waste (MSW), commercial and industrial waste (C&IW), and construction and demolition waste (C&D). In most high-income countries, C&IW is usually utilized in waste incineration plants after separating recyclable and inert materials from it (Ragoßnig et al., 2009). Incineration of C&IW is economically and environmentally challenging due to its heterogeneous characteristics (Donahue, 2018). In the majority of middle-income or low-income countries, C&IW are usually disposed of at landfills, causing potential environmental damage. Diverting C&IW from incineration plants and landfills to cement plants could be environmentally and economically feasible and an easy way also for low-income countries to reduce landfilling and increase recovery of waste without large investments. To determine the impact of using SRF on whole systems, including cement production and waste management systems, it is important to conduct a credible analysis on SRF utilization in cement production.

Life cycle assessment (LCA) is a method for quantifying the potential environmental impact of a product or service throughout its life cycle (International Organization for Standardization, 2006). The environmental impact of utilizing alternative fuel in cement plants has already been investigated as part of several LCA studies. Examples of these studies include C&D wood waste (Hossain et al., 2017), tyre derived fuel (TDF), biological sludge (BS), refused derived fuel (RDF), and a mixture of TDF, BS, and RDF (Georgiopoulou and Lyberatos, 2018), C&IW (Helftewes et al., 2012), and C&D wood waste, asphalt shingles, railway ties, and plastics (Zhang and Mabee, 2016). Güereca et al. (2015) have studied the environmental impact of utilizing RDF (which is produced from MSW) in cement while expanding the system boundary to the landfilling of waste. All the aforementioned studies found that waste-derived fuel utilization as an alternative can have significant impact on GHG emissions reduction in the cement production process. However, none of the LCA studies have analysed the GWP impact of utilizing C&IW in the cement industry while also focusing on the waste management system which is eventually affected by the utilization of C&IW as fuel in the cement production process.

Waste can be treated in the cement industry or waste incineration plants. Also, in many parts of the world, landfill is still the ultimate option for waste treatment. Energy can be recovered from waste incineration or the landfilling process and used for replacing energy, which is produced from other sources. Therefore, while using waste in the cement industry, it is important to investigate the impact when waste is not used for energy recovery and thus no energy is replaced. This study combines both the cement industry and affected waste management system together, while considering SRF which is derived from C&IW as fuel in the cement industry.

One of the main objectives of this study is to quantify the change in global warming potential (GWP) of cement production when conventional fuels are replaced with SRF in a cement plant. Since in this study, the alternative fuel is SRF, this means that there are also changes in the associated waste management system that need to be considered. Therefore, this study also investigates, the GWP impact of the affected waste management system.

## Materials and methods

The LCA in this study was conducted in accordance with ISO 14040 standards (International Organization for Standardization, 2006), which defines four major phases: (a) goal and scope definition; (b) inventory analysis; (c) impact assessment; and (d) interpretation. The first three phases are presented below. The interpretation of this LCA study, which includes results, sensitivity analysis, and recommendations, is presented in a separate results analysis and discussion section. This study is partially based on the work conducted by Khan (2018). However, the LCA conducted by Khan (2018) did not assess the environmental impact of C&IW management when utilizing SRF in cement plants.

### Goal and scope definition

Finnsementti’s cement production (in Lappeenranta factory) is used as a case for this study. According to Finnsementti (2017), the cement plant in Lappeenranta uses a dry long rotary kiln with a four-stage cyclone pre-heater followed by a pre-calciner. The study has been conducted based on fiscal year 2007 and 2016 cement production data, where SRF use has been increased from zero to 53%. Furthermore, the company intends to increase the share of SRF in its fuel usage. It has been estimated that 80% of fuel energy content is the technical maximum for SRF use. The aim of using real measured data is to show that the proposed changes are realistic and that similar practice could be done in other parts of the world.

The goal of the study is to determine the change in environmental impact when replacing conventional fuels with SRF in the cement production process as well as diverting C&IW from waste incineration and landfills to cement plants. In this study, it was estimated that to supply enough SRF to replace 80% of the fuel energy needed to produce 1 Mt of cement in the case plant, 194 kg of C&IW would be needed. That is why the functional unit of this study is the production of 1 Mt of ordinary Portland cement (OPC) and treatment of 194 kg C&IW.

A cradle-to-gate approach was selected, and the LCA calculations were performed using GaBi 8.0 software (Thinkstep, 2018). Impact assessment was conducted using the CML2015 method (version: January 2016). The quantity of nitrogen oxides (NOx), sulphur oxides (Sox), and other emissions reduction due to the utilization of SRF in the plant is uncertain from plant operation data, because other technical changes have been initiated related to the emission control in the same time period when SRF use has been increased. There has been continuous improvement in the air pollution control in the plant partially based on the experiences of SRF usage in another cement plant, which is located in Parainen, Finland. To control various emissions such as particulate matter, NOx, and SOx, the plant has installed electrostatic precipitators, fabric filters, selective non-catalytic reduction, low-NOx burners and a new furnace. Since, in the exception with CO_2_ emission, the reduction of the emissions of NOx, SOx, and other emissions due to the utilization of SRF is uncertain, therefore, NOx, SOx, and other emissions were excluded from this study. That is why the impact category evaluated in this study is GWP, which represents an important area of environmental concern for cement production. The possible changes in NOx and SOx emissions would affect acidification and eutrophication impact categories but the relation between NOx and SOx emission with SRF utilization could not be established. In addition, the environmental requirements in Finland dictate that the NOx and SOx emissions remain within the allowed limits and these emissions can be controlled with air pollution devices which were installed limiting the possible environmental impact changes.

### System boundary and scenarios

**Figure 1.**
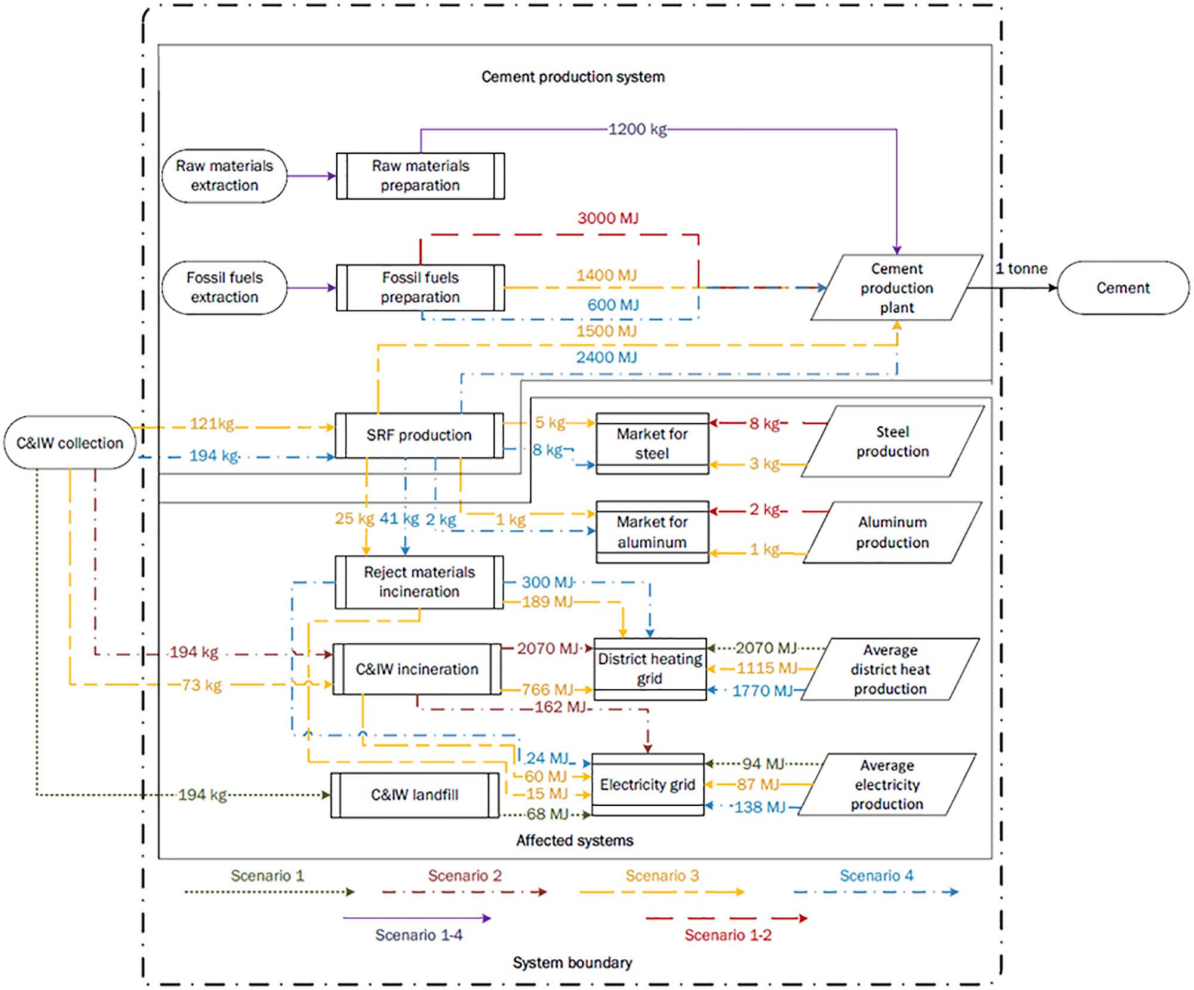
System boundary and mass and energy flows in studied scenarios.

The system boundary of this LCA model is illustrated in Figure 1. The system boundary is divided into two sections: cement production system; and affected waste management systems. Assessment started from the collection of C&IW and the extraction of raw materials and fossil fuels (FFs). The cement production system includes raw materials and FF extraction, SRF production from C&IW, and transportation of raw materials, FFs, and SRF to the cement plant. Cement production systems also include unit operations of the cement production system, such as grinding and mixing of raw materials, calcination process, and clinker cooling, grinding, and mixing with additives. Furthermore, this study also includes electricity consumption during preparations of raw materials, FFs and other plant operations, and thermal energy consumption for clinker production. The system boundary ends with the production of the cement. The potential environmental impacts from packaging, transportation, utilization, and cement product end-of-life were excluded, since the emissions from these processes are considered to be equal for all the scenarios.

The affected systems include the transportation and treatment of C&IW in landfills or incineration plants, recycling of by-products of the system, such as steel and aluminium from the SRF production process, electricity and district heating from waste incineration, and electricity from landfill gas (LFG). In addition, the affected systems include steel and aluminium production from recycling systems, average district heating production from Finnish district heating production mix in 2017, and electricity production from the Finnish electricity production mix in 2017.

According to Chen et al. (2010), waste can be considered as an environmental burden free from the previous life cycle phases (material acquisition and refining, manufacturing, and use of products) when the environmental burdens during its production phases are allocated to the products. Thus, in this study, it is assumed that C&IW and other waste material such as waste oil entered into the system without any environmental burden. In addition, several raw materials – for example, furnace slag, iron (roll scale), and fly ash – are also considered to be waste material. Hence, only the environmental burdens for transporting those aforementioned waste materials, raw materials, and additives are included in this analysis.

Four scenarios were analysed with varying C&IW treatment methods and SRF share in the thermal energy consumption mix during clinker production. Calculations were made using the system expansion method; therefore, no emission displacement resulted from the process. Instead, in all the scenarios, the same amount of electricity, heat, and materials go into and leave the system boundary.

The maximum amount of C&IW treated occurs in Scenario 4 (SC 4), where 80% of thermal energy is supplied by SRF. That same quantity of C&IW has to be treated in all other scenarios. Since SC 4 has the highest mass of C&IW directed to SRF production at the plant, this scenario also has the highest amount of metal recovery. As a result, the amount of metal production for the remaining three scenarios must be equal to SC 4. Those scenarios with lesser amounts of metal output have to obtain metal supply from external sources, which are assumed to be recycled aluminium and steel. The CO_2_ emission factors of recycled aluminium and steel are presented in the Online Supplementary Table 1. Energy recovered from C&IW incineration in SC 2 can be used for energy replacement. Since, SC 1, SC 3, and SC 4 do not have an equal amount of energy supply to SC 2, these scenarios are required to have energy supply from other sources.

1. **Scenario 1 (SC 1):** In this scenario, 100% of the thermal energy consumed during clinker production is supplied from FF sources, which comprised 47% from coal, 47% from petroleum coke, and 6% from waste oil. The collected 194 kg C&IW is considered to be disposed of at landfill sites, where LFG is collected and used for electricity production.
2. **Scenario 2 (SC 2):** Similar to SC 1, an equal quantity and share of FFs are used during clinker production in SC 2. However, instead of landfill disposal, the untreated C&IW is directed to waste incineration plants for recovering heat and electricity.
3. **Scenario 3 (SC 3):** SRF supplies 50% of total required thermal energy for cement production. The remaining energy requirements are met through FFs, which includes 37% from petroleum coke, 4% from coal, 5.5% from waste oil, and 0.5% from light fuel oil (LFO). In this scenario, 121 kg of C&IW (which is equal to 75% of total energy content of C&IW) is used for SRF production, whereas the rest of the 73 kg of C&IW is treated in the waste incineration plant for recovering energy.
4. **Scenario 4 (SC 4):** In this scenario, SRF provides 80% of the total thermal energy. The remaining energy is supplied from petroleum coke. In SC 4, all the collected C&IW is used for SRF production.

### Inventory data and calculation principles

The data used in this study are based on cement production from Finnsementti’s Lappeenranta plant. Since some of the detailed data from Finnsementti Oy are deemed to be confidential, only the aggregated data from the cement production process are presented in this research paper. Plant data are used to analyse the environmental impact when the SRF share in the thermal energy supply is 0%, 50%, and 80%. A 100% replacement of conventional fuels by SRF in the cement production process is not technically or economically viable due to several challenges. Among the challenges, managing the emission of sulphur dioxide, chlorine, NO_*x*_, and carbon monoxide, possible blockages in the heat exchange system, control of pre-calciner operations, and ensuring the quality of the produced cement are considered to be the most crucial (Chinyama, 2011).

#### Raw materials acquisition

Finnsementti utilizes on average 1200 kg of raw materials for 1 Mt of cement production. The utilized raw materials for cement production can be divided into five major components to provide calcium oxides, aluminium oxides, silica, ferrous oxides, and calcium sulphate (Huntzinger and Eatmon, 2009). In this study, raw materials are mainly constituted by limestone (81%), followed by fly ash (8%), blast furnace slag (4%), gypsum (3%), and mineral waste (2%). The remaining raw material components include diabase, bauxite, and iron (roll scale). In this study gypsum, and mineral waste were used as admixture, which were mixed with clinker to produce cement. The GHG emissions from raw materials acquisition, RM_GHG_ (kg CO_2_, eq./ Mt cement), is calculated using Equation (1). CO_2_ emission from each of the raw materials preparation has been collected from GaBi thinkstep database (see Online Supplementary Table 2):

(1) $$RM_{GHG}=\sum_{i}^{n}\left(m_{RM}*w_{RM,i}*CE_{RM,i}\right)$$

where

*m_RM_* = total mass of raw materials per Mt cement (kg Mt^−1^ cement),

*w_RM,i_* = share of raw material i (kg raw material i (kg total raw material)^−1^), and

*CE_RM,i_* = CO_2_ emission from raw material i preparation (kg CO_2_, eq. (kg raw material i)^−1^).

The emissions from thermal energy consumption are caused by fuel preparation and fuel combustion. For calculating the emissions from fuel preparation, the CO_2_ emission factor from the preparation of FFs, CEF_p_ (kg CO_2_, eq. kg^−1^) such as coal, petroleum coke, and LFO were collected from the GaBi thinkstep database (see Online Supplementary Table 3). The total GHG emissions of fuel preparation, FP_GHG_ (kg CO_2_, eq. (Mt cement)^−1^), can be calculated by Equation (2), utilizing CEF_p_ and required mass of fuels which is presented in Online Supplementary Table 4:

(2) $$FP_{GHG}=\frac{\sum_{i}^{n}\left(m_{F,i}*CEF_{p,i}\right)}{1000}$$

where

*m_F,i_ =* mass of fuel i per mass of cement (kg (Mt cement)^−1^),

*CEF_p,i_ =* CO_2_ emission factor FF i preparation (kg CO_2_, eq.(kg fuel i)^−1^).

The emissions from fuel combustion are caused by the fossil carbon content of the fuels. The CO_2_ emission factor of SRF combustion per energy content, CEF_e,SRF_ (g CO_2_, eq. MJ^−1^) is calculated based on the compositions as well as non-biogenic carbon content of the waste fractions, which are presented in Online Supplementary Table 5. The mass of non-biogenic carbon per mass of SRF, m_Cnb,SRF_ (g C (kg SRF)^−1^), is calculated by Equation (3):

(3) $$m_{Cnb,SRF}=\sum_{i}^{n}\left(w_{SRF,i}*TS_{SRF,i}*C_{SRF,i}*Cnb_{SRF,i}\right)$$

where

*w_SRF,i_ =* share of SRF component i (kg SRF component i (kg SRF)^−1^),

*TS_SRF,i_ =* share of total solid in the SRF component i (kg total solid (kg SRF component i)^−1^),

*C_SRF,i_ =* share of C in the total solid of the SRF component i (g C (kg total solid)^−1^), and

*Cnb_SRF,i_ =* share of non-biogenic C in the total C of the SRF component i (g non-biogenic C/(g C)^−1^).

The mass of carbon dioxide per mass of SRF (g CO_2_ (kg SRF)^−1^) is calculated by Equation (4):

(4) $$\frac{m_{CO_{2}}}{m_{SRF}}=m_{Cnb,SRF}*\frac{{\left(n*M\right)}_{CO2}}{{\left(n*M\right)}_{C}}$$

where

*n =* molar amount (mol), and

*M =* molar mass (g mol^−1^).

The emission factor of SRF combustion per energy content of SRF, CEF_e,SRF_ (g CO_2_ MJ^−1^), can be calculated with Equation (5):

(5) $$CEF_{e,SRF}=\frac{\frac{m_{CO_{2}}}{m_{SRF}}}{LHV_{ar,SRF}}$$

where

*LHV_ar,SRF_ =* lower heating value as received of SRF (MJ kg^−1^).

The total fuel energy need was considered to be 3000 MJ per Mt of cement. The quantity of fuel required in the scenarios depends on the LHVar of the fuels. Online Supplementary Table 6 presents the LHVar of utilized fuels along with their CO_2_ emission factor. Based on the thermal energy requirement for 1 Mt of cement, SC 1 and SC 2 utilized 106 kg of fuel. In SC 3, fuel utilization was increased to 125 kg for 1 Mt of cement because the SRF share in the thermal energy supply was increased to 53% as well as SRF has lower LHVar compared to the FFs used in this study. The quantity of utilized fuel was increased even further to 139 kg in SC 4 when the SRF share was increased to 80%.

The total amount of GHG emissions from fuel combustion, FC_GHG_ (k*g* CO_2_, eq. (Mt cement)^−1^), depends on the LHVar and CO_2_ emission factor of the fuels and can be calculated with Equation (6):

(6) $$FC_{GHG}=\frac{\sum_{i}^{n}\left(TE*w_{TE,i}*CEF_{e,i}\right)}{1000}$$

where

*TE =* required thermal energy (MJ (Mt cement)^−1^),

*w_TE,i_ =* share of fuel i (MJ (MJ thermal energy)^−1^), and

*CEF_e,I_ =* CO_2_ emission factor of fuel i (g CO_2_, eq. MJ^−1^).

The overall GHG emissions from thermal energy consumption, TE_GHG_ (kg CO_2_, eq. (Mt cement)^−1^), is determined by Equation (7):

(7) $$TE_{GHG}=FP_{GHG}+FC_{GHG}$$

#### Calcination process

In this study, it was assumed that calcium carbonate and magnesium carbonate from limestone was completely decomposed due to the high temperature (1450°C) and lengthy burning time. The amount of GHG emissions from the calcination process depends on several factors, such as the amount of clinker in the cement and the share of calcium oxide (CaO) and magnesium oxide (MgO) in the clinker. In this study, the clinker to cement ratio was 80%, whereas the mass share of CaO and MgO in clinker were 65% and 3%, respectively. The remaining portion was composed mainly of silicon dioxide and, to a smaller extent, aluminium oxide and iron oxide. The amount of GHG emissions produced by the calcination process, CP_GHG_ (kg CO_2_, eq. (Mt cement)^−1^), was calculated to be 438 kg CO_2_, eq. (Mt of cement)^−1^ using Equation (8):

(8) $$CP_{GHG}=w_{cl}*\left(w_{CaO}*\frac{M_{CO_{2}}}{M_{CaO}}+w_{MgO}*\frac{M_{CO_{2}}}{M_{MgO}}\right)$$

where

*w_cl_ =* share of clinker in cement (kg clinker (kg cement)^−1^),

*w_CaO_ =* share of CaO in the clinker (kg CaO (kg clinker)^−1^),

*w_MgO_ =* share of MgO in the clinker (kg MgO (kg clinker)^−1^),

*M_CaO_ =* molar mass of CaO (kg kmole^−1^), and

*M_MgO_ =* molar mass of MgO (kg kmole^−1^).

#### Electricity consumption

In this study, electricity is consumed in the cement production process for raw materials preparation, coal crushing, clinker production process, finishing process, as well as in the SRF production process. Electricity consumption for the preparation of raw materials is included in the roll mill operation, smashing machines, exhaust fans and grate coolers, and kiln hood operations during the clinker production process. During the finishing process, electricity is consumed for grinding of clinker, gypsum and different admixtures, such as fly ash and furnace slag. This study included the electricity production mix of Finland in 2017, which comprised 10% coal, 4% natural gas, 13% biofuels, 26% nuclear, 17% hydro, 5% wind and the rest of the 25% was imported from Nordic countries, Russia and Estonia (IEA, 2020a) . The CO_2_ emission factor of electricity production was 158 kg CO_2_, eq. per MWh (Motiva, 2019).

#### C&IW management

The composition of C&IW along with the reject materials which is produced from SRF production process determined by Nasrullah et al. (2014) is used and presented in Online Supplementary Table 7. The data were relevant for this study, because Nasrullah et al. (2014) have analysed C&IW in Finland. According to Nasrullah et al. (2014) C&IW and reject materials contain a significant share of paper and cardboard, soft and hard plastic waste, followed by textiles and wood.

In SC 3 and SC 4, SRF is produced from C&IW, after passing through the required treatment processes such as shredding, screening, metal separation, air classification, near-infrared sorting, and the dust extraction system (Nasrullah et al., 2014) (see Online Supplementary Figure 1). In addition to SRF, fine and heavy fractions, metals (ferrous and nonferrous), and reject materials are also produced from the SRF production process. According to Nasrullah et al. (2014), 62% of C&IW mass is converted in to SRF, whereas 21% of the C&IW mass is rejected, 12% is sorted out as fine and heavy fractions, and the rest of the 5% is collected as ferrous and nonferrous metals. The energy conversion from C&IW to SRF was considered as 75% (Nasrullah et al., 2014), which means that SRF, which is 62% of the mass of C&IW, has 75% of the energy content of the C&IW from which it is produced. The LHVar of C&IW was calculated to be 17 MJ kg^−1^ using the heating value of SRF which was obtained from Finnsementti (2017). Reject materials were assumed to be used for energy production in waste-to-energy plants. The LHVar of reject materials (11 MJ kg^−1^) was obtained from Nasrullah et al. (2014). The ferrous and nonferrous metals were recovered, and the fine and heavy fractions were disposed of in the landfill without any energy recovery. The annual energy efficiency of waste-to-energy plants was assumed to be 69%, including thermal energy production efficiency of 64% and electric efficiency of 5% (Anttila, 2011).

The CO_2_ emission factor of C&IW and reject materials are calculated based on the composition and the non-biogenic carbon content of the waste fractions (see Online Supplementary Tables 8 and 9). The calculated CO_2_ emission factor of C&IW was 49 g CO_2_, eq. MJ^−1^ and for reject material, it was 42 g CO_2_, eq. MJ^−1^. It was mentioned in the “System boundary and scenarios” subsection that all the scenarios should have the same amount of energy output from the system. If a scenario does not have an equal amount of energy output from the system, energy from outside sources is required. In this study, it was assumed to be the electricity and district heating production mix in Finland in 2017. The average emission of Finnish district heat production has been 164 kg CO_2_, eq MWh^−1^ (Motiva, 2019).

In SC 1, the collected C&IW is disposed of at landfill, where C&IW is decomposed into LFG, which included methane (CH_4_), CO_2_ and other compounds produced through the anaerobic digestion process. C&IW landfilling emission data were obtained from the GaBi database and 28% of the LFG was assumed to be directed to electricity production, 22% to flare, and 49% released to the atmosphere (Thinkstep, 2018). The calculated GHG emissions from landfilling of 194 kg of C&IW are the equivalent of 142 kg CO_2_.

#### Transportation system of the model

Depending on the import location of raw materials, fuels and C&IW, two types of transportation systems are used in this model: heavy fuel oil-powered ship; and diesel-powered truck. Among the raw materials, gypsum, blast furnace slag, and bauxite are transported using ship and truck, whereas the other raw materials such as fly ash are transported by truck. Coal and petroleum coke are transported by ship and truck, when SRF, LFO, and waste fuel oil are transported by truck. Due to the confidentiality issue, the transportation distance for raw materials and fuels is not presented in this study. The distance of transporting C&IW to the SRF plant as well as waste incineration plant and landfill was assumed to be 100 km. The emissions from transportation of the raw materials and fuels are acquired from the GaBi Thinkstep database.

### Inventory data for sensitivity analysis

Thermal energy consumption is one of the major sources of GWP production in cement production. Therefore, thermal energy consumption is included in the sensitivity analysis to determine the GWP impact when thermal energy requirement is changed during the calcination process. The thermal energy requirement of the Finnsementti plant in 2016 (3000 MJ (Mt cement)^−1^) was compared to requirements in 2007 (3400 MJ (Mts cement)^−1^).

There is a straightforward relationship between the proportion of clinker in the cement and the amount of GHG emissions produced by the calcination process. The need of thermal energy for 1 Mt of cement production also depends on the clinker ratio in cement. That is why the influence of clinker share on total GHG emissions from the cement production process is investigated as part of the sensitivity analysis in this study. It is assumed that in 2050 the proportion of clinker in cement production will be on average 70% (CEMBUREAU, 2013). Therefore, as part of the sensitivity analysis, cement production with a clinker share of 70% is compared with baseline scenarios where the clinker share is 80%.

Wood fuel is the largest source (33%) of district heating in Finland, followed by hard coal (23%) (Statistics Finland, 2018). Therefore, the sensitivity analysis also includes the GWP impact of producing the additional heat by hard coal and solid biomass.

## Results analysis and discussion

### GWP of the implemented scenarios

**Figure 2.**
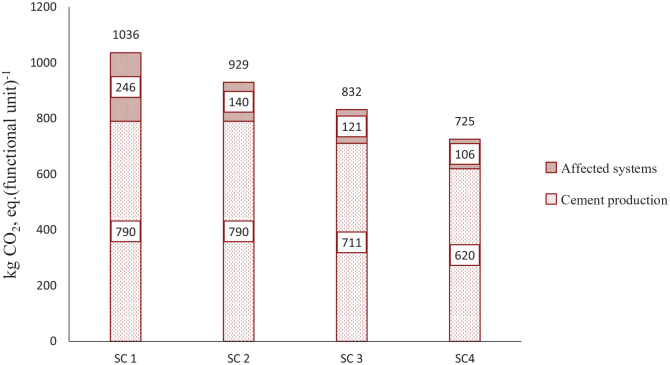
Global warming potential impact of utilizing solid recovered fuel (SRF) in the cement industry, including the affected waste management system caused by the utilization of SRF.

The results in Figure 2 summarize the GWP impact of both the cement production process and affected waste management systems. The total GWP is reduced from 1036 kg CO_2_, eq. (functional unit)^−1^ in SC 1 to 725 kg CO_2_, eq. (functional unit)^−1^ in SC 4. GWP from cement production remains unchanged between SC 1 and SC 2, because in both of the scenarios, equal amounts and share of fuels have been used. However, due to the consideration of different C&IW management methods, SC 1 generates more than 100 kg CO_2_, eq. (functional unit of emissions from affected system)^−1^ than SC 2. Furthermore, by applying SRF in the kiln, SC 3 and SC 4 experience reduction in both the cement production process and affected system.

### Relative contribution on GWP in cement production

**Figure 3.**
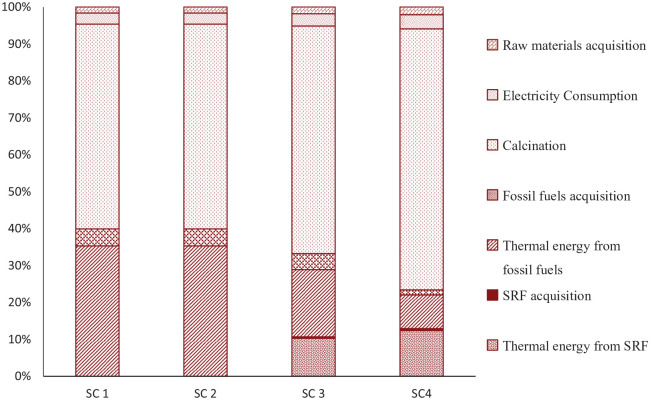
Relative contribution on global warming potential within the cement production system.

In SC 1 and SC 2, the cement production process generates 790 kg CO_2_, eq. (functional unit)^−1^, which is mainly comprises emissions from CO_2_ release during calcination, 55%, and fuel combustion during the calcination process, 35%. Figure 3 presents the relative share on GWP from the cement production system and more detailed information can be found from the Online Supplementary Table 10. Calcination and fuel combustion together generate 90% of total GHG emissions from cement production, which is consistent with the published figure from World Business Council for Sustainable Development (WBCSD) (2005). When 53% of total FFs are replaced by SRF, GHG emissions from cement production are reduced by 10% compared to SC 1 and SC 2. This result is similar to the study conducted by García-Gusano et al. (2015) , where GHG emissions were reduced by 9% by altering 50% FFs with alternative fuels. In this study, it is found that GHG emissions are reduced even further by 22% compared to SC 1 and SC 2, when the SRF share in the fuel energy reached to 80%. GHG emissions from acquisition of raw materials (2% in all scenarios) and FFs (1% to 5% in all scenarios) including transportation have low impact. In addition, electricity consumption during plant operation of cement production has also quite low impact (3% to 4% in all scenarios).

The GHG emissions reduction for utilizing SRF in the cement production process suggests that SRF is an effective alternative fuel for reducing GHG emissions from the cement industry. The results also demonstrate that GHG emissions reduction increases with the increase of SRF share in the thermal energy. The emission reduction primarily depends on the biogenic C share of SRF. SRF containing a higher share of components such as paper and wood would reduce more GHG emissions compared to SRF with a higher share of plastics, because paper and wood contain more biogenic C compared to plastic (Kim et al., 2016). However, lower amounts of plastic and higher amounts of paper and wood in SRF would reduce the LHVar of SRF, because plastic has higher LHVar (35–40 MJ kg^−1^) (Panda et al., 2010) than paper and wood (15–16 MJ kg^−1^) (Strazza et al., 2011). If a lower LHVar of SRF is utilized in the cement industry, it can lead to a large quantity of SRF consumption to meet the energy demand and thus increase the GHG emissions from SRF acquisition as well as from the cement production process, which is evident from the study conducted by Georgiopoulou and Lyberatos (2018).

### Impact of utilizing SRF on affected waste management system

The SRF utilization in cement plants has a significant impact on the GWP of the affected waste management system, which can be seen from Table 1. GHG emissions from incineration of C&IW in SC 2 are close to landfilling of C&IW in SC 1, because the studied C&IW contains more than 50% plastic, foam, metal, glass, stones and fines. In the landfilling process, organic matter decomposes to GHGs such as CH_4_, and CO_2_. Since plastic is hard to break down and degrades over a very long period of time, it hardly contributes to the GHG emissions. On the contrary, plastic waste incineration generates GHG emissions. In SC 3, 80% of the emission results from C&IW incineration, and the rest from the incineration of rejects from SRF production. In SC 4, emission from incineration comes only from reject incineration. The result of the study shows that in C&IW management, 80% SRF utilization in the cement industry reduces up to 85% of GHG emissions from SC 1 and 84% from SC 2. However, taking the overall affected system into consideration, SC 4 generates 57% less emission from SC 1 and 24% less emission from SC 2, which is primarily due to the heat supply from outside sources.

**Table 1.** Global warming potential impact from an affected waste management system for utilizing solid recovered fuel in a cement plant.

Scenario	Commercial and industrial waste management			Heat addition to the system	Electricity addition to the system	Metal addition to the system	Unit
	Landfill	Incineration	Transportation				
SC 1	142		1	93	4	5	kg CO_2_, eq. (functional unit)^−1^
SC 2		133	1			5	
SC 3		51	2	53	11	4	
SC4		19	2	80	5		

Average GWP of global cement production was accounted 800–1000 kg CO_2_, eq. (Mt cement)^−1^ (Chen et al., 2010; Li et al., 2014; Valderrama et al., 2012). SC 4 of this study shows that the GWP from cement production could be reduced by 22–38% from average emission, which is in global scale 0.7–1.5 billion Mts in a year. This study shows that supplying 80% fuel energy by SRF in the global cement industry would require about 0.9 billion Mts of C&IW. The mass of global C&IW is uncertain but according to the World Bank, in 2016, 2 billion Mts of MSW were produced worldwide, out of which 40% was mismanaged (Ellis, 2018). In some regions the C&IW could be included also in MSW mass. There is a strong link between waste and climate change. Waste accounts for 5% of global GHG emissions, which is mainly due to the open dumping and landfilling of waste (Ellis, 2018). Instead of landfilling, wastes are valorized through incineration in most of the high-income countries. Waste treatment in waste incineration plants is economically challenging due to the establishment of new plant, operation and management, and a proper emissions control system. Therefore, waste burning in waste incineration plants might not be a proper solution for low-income countries, especially for those who have existing budget problems. In this case, rather than building a new waste incineration plant, it would be cheaper to utilize the wastes in existing cement plants. However, one of the biggest challenges of utilizing SRF in the cement industry is the lower LHVar of waste due to the improper waste management system. Therefore, it is important to embrace an integrated solid waste management system, where combustible parts of wastes would be separated and collected efficiently.

### Sensitivity analysis

**Figure 4.**
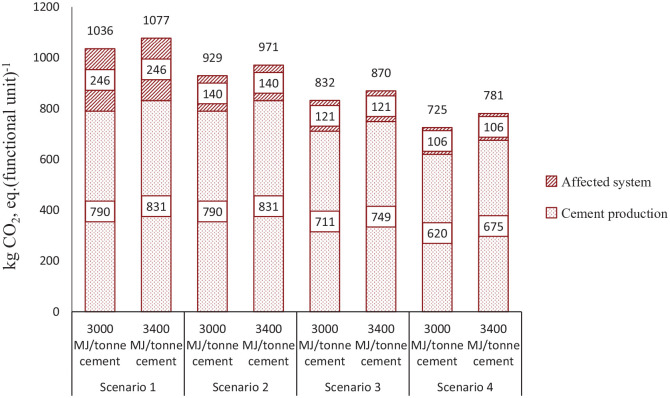
Comparison of the greenhouse gas emissions with higher thermal energy requirement scenarios.

The GHG emissions from cement production processes, such as fuel acquisition, preparation, and transportation are related to the per unit thermal energy consumption in the plant. When the thermal energy requirement is changed, GHG emissions from those processes change simultaneously. Since the functional unit is unchanged, the amount of energy supply from SRF is also unchanged. However, the fuel energy share changes in SC 3 and SC 4. The fuel energy share for supplying 3400 MJ of thermal energy is estimated to be 47% SRF, 42% petroleum coke, 4% coal, 6% waste oil, and 1% LFO in SC 3. In SC 4, the estimated fuel energy share is 70% SRF and 30% petroleum coke. The sensitivity analysis result on increased energy demand in cement production (see Figure 4) shows that GHG emissions increased by 3% to 6% in all the scenarios, when the energy demand increases by 13% from 3000 MJ.

Thermal energy consumption in the cement plant depends on the clinker share in the cement. The sensitivity analysis revealed that thermal energy consumption for 1 Mt of cement decreased from 3000 MJ to 2600 MJ when clinker share in the cement was reduced from 80% to 70%. Since the thermal energy need is reduced and functional unit remains unchanged, the fuel energy share in thermal energy is changed to 62% for SRF, 31% for petroleum coke, 3% for coal, and 4% for waste oil in SC 3. In SC 4, the estimated fuel energy share is 92% SRF and 8% petroleum coke. It was discussed earlier that obtaining more than 80% of fuel energy from SRF was economically and technically challenging. Therefore, 92% SRF in thermal energy share requires further technical development and budget for appropriate plant design.

**Figure 5.**
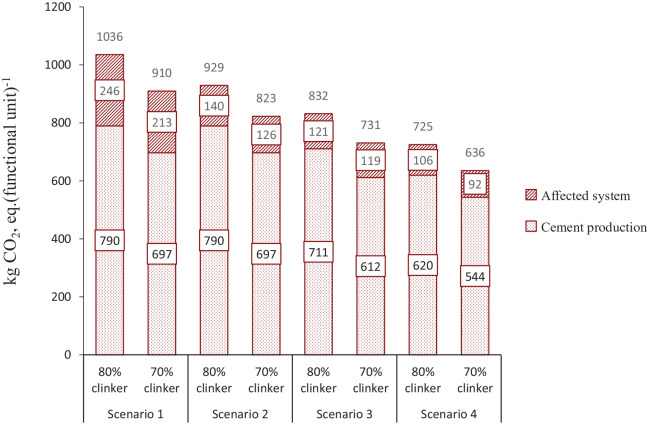
Comparing greenhouse gas emissions resulted from two different shares of clinker.

The results of the sensitivity analysis in Figure 5 show that reducing clinker in cement can cause GHG emissions reduction not only from the calcination process but also from the thermal energy consumption process and all other processes related to the thermal energy consumption process. Results revealed that 13% reduction in clinker share resulted in reducing GHG emissions by 9% to 14% in all the scenarios. Fly ash, blast furnace slag, natural pozzolans, silica fume, and limestone powder can be used as substituent for clinker (CEMBUREAU, 2013). European cement industries must follow the European cement standard EN 197-1, which identifies 27 common types of cements containing a clinker-to-cement ratio that ranges from 5% to 95% (CEMBUREAU, 2013). Thus, reducing the clinker share in the cement might change the type of cement and have an impact on market acceptance. Nonetheless, the utilization of substitute clinker materials relies on the availability of those materials (CEMBUREAU, 2013). For example, the availability of fly ash is dependent on coal-fired power plants and the supply of blast furnace slag depends on pig iron–steel production.

**Figure 6.**
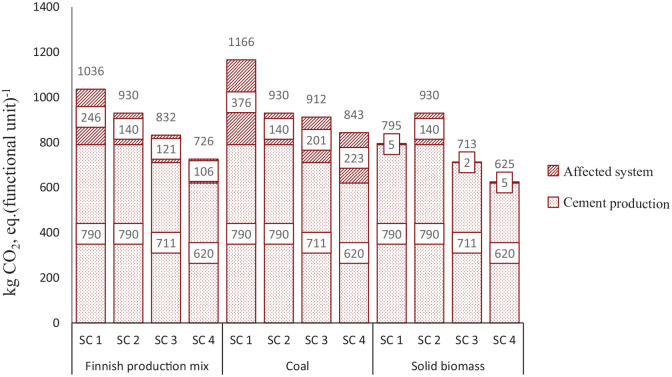
Changes in greenhouse gas emissions due to the differences in heat sources.

In this study, GHG emissions from an affected system significantly relies on the addition of electricity and district heat to the system and is more affected by the addition of district heat. The results presented in Figure 6 reveal that total GHG emissions in the scenarios can be affected by altering the additional district heat source. Results reveal that in SC 1, SC 3, and SC 4, emissions from an affected system increase when the additional heat is produced from coal and decreases when it is sourced from solid biomass. Directing C&IW to an incineration plant is more preferable than landfill, when the thermal energy is added from the Finnish production mix or had coal. On the contrary, it is not environmentally beneficial to burn C&IW in incineration plants when the additional heat is sourced from solid biomass. Thus, the more renewable are the sources of heat in the region, the more sustainable it is to use the SRF as a fuel in the cement production.

To sum up, this study shows that, the best option to achieve highest environmental benefit from cement production as well as the system affected by the fuel utilization in the cement industry is to utilize 80% SRF in the cement industry. This also needs a thermal energy requirement of 3000 MJ (Mt cement)^−1^ and a 70% clinker to cement ratio. The emission reduction for using SRF could be gained in a case where a dry rotary kiln with pre-heater and pre-calciner are used. A wet rotary kiln requires more thermal energy than a dry rotary kiln, which may lead to more fuel consumption and thus generate more emissions. Also, locally produced SRF should be used to minimize emissions from transportation. Since this study is limited to GWP impact, a further assessment on other impact categories should be conducted.

## Conclusions

The change in GWP of both the cement production and affected waste management systems when introducing SRF to replace conventional fuels in cement plants was analysed using LCA. The study was based on real plant data, where four scenarios were established with a varying share of SRF in the thermal energy consumption mix of the cement plant. According to the results, SRF can play an important role in reducing the GWP of the cement production system, while also reducing the GWP of the affected system by diverting C&IW from landfills or waste incineration plants to cement plants. The GWP of both systems decreased along with the increase of SRF share from the thermal energy consumption mix. Sensitivity analysis demonstrated that the environmental benefits from utilizing SRF in cement plants can be increased by lowering the clinker-to-cement ratio and thermal energy requirement for clinker production.

Despite SRF utilization having significant impact on the reduction of the GWP of cement production and C&IW management systems, certain aspects need to be borne in mind. First, LHVar of the SRF depends on the composition of waste, which also affects the CO_2_ emission factor. Waste fractions with high fossil carbon content usually have higher LHVar compared to fractions with higher biogenic carbon content, meaning that SRF with higher LHVar also has higher CO_2_ emission factor and vice versa. On the other hand, lower LHVar means that more SRF is required to provide an equal amount of fuel energy as with SRF with higher LHVar, which lowers the benefit of a lower CO_2_ emission factor. Second, for middle-income and low-income countries to achieve environmental benefits from SRF utilization similar to those in high-income countries, an integrated waste management system, including waste collection with source separation practice, should be developed.

## Supplemental Material

sj-pdf-1-wmr-10.1177_0734242X20978277 – Supplemental material for Impact of utilizing solid recovered fuel on the global warming potential of cement production and waste management system: A life cycle assessment approach Click here for additional data file. Supplemental material, sj-pdf-1-wmr-10.1177_0734242X20978277 for Impact of utilizing solid recovered fuel on the global warming potential of cement production and waste management system: A life cycle assessment approach by Md Musharof Hussain Khan, Jouni Havukainen and Mika Horttanainen in Waste Management & Research
