# Supplementary material for: Impact of utilizing solid recovered fuel on the global warming potential of cement production and waste management system: A life cycle assessment approach
Source: Waste Manag Res. 2020 Dec 25;39(4):561–72. doi: 10.1177/0734242X20978277 (PMC7952896; doi:10.1177/0734242X20978277)
Supplement: sj-pdf-1-wmr-10.1177_0734242X20978277 – Supplemental material for Impact of utilizing solid recovered fuel on the global warming potential of cement production and waste management system: A life cycle assessment approach [file sj-pdf-1-wmr-10.1177_0734242X20978277.pdf]

Supplementary Material

Table 1. CO_2 emission factor of metals (CEF_{metal}).

Metals	CEF_{metal} (kg CO_2, eq./kg) (virgin material)	CEF (kg CO_2, eq./kg) (recycled material)
Aluminum	7.100 (Thinkstep,2018)	a 0.820
Steel	2.320 (Thinkstep, 2018)	b 0.660

[a] 12% recycled aluminum [b] 29% recycled steel (cBalance Solutions Hub, 2013)

Table 2. CO_2 emission factor of raw materials acquisition (CEF_{ra}).

Components	CEF_{ra} (kg CO_2, eq./kg)	Reference
Limestone	0.005	Thinkstep,2018
Bauxite	0.003	Thinkstep,2018
Diabase	0.003	Thinkstep,2018
Silica sand	0.036	Thinkstep,2018
Gypsum	0.090	Thinkstep,2018
Petroleum coke	0.312	Thinkstep,2018
Hard coal	0.122	Thinkstep,2018
LFO	0.318	Thinkstep,2018

Table 3. CO_2 emission factor of fossil fuel preparation (CEF_p).

Fossil fuel	CEF_p (kg CO_2, eq./kg fuel)	Reference
Petroleum coke	0.312	Thinkstep,2018
Hard coal	0.122	Thinkstep,2018
LFO	0.318	Thinkstep,2018

Table 4. Mass of fuel utilization for one tonne of cement production (unit: kg/ tonne cement).

Fuel	SC 1	SC 2	SC 3	SC 4
petroleum coke	44	44	35	19
coal	56	56	5	0
waste oil	6	6	6	0

LFO	0	0	0	0
SRF	0	0	80	120
Total	106	106	125	139

Table 5. Total solid and non-biogenic carbon of SRF components.

SRF	share	Total solid content (TS)	Total share of carbon (C)	Non-biogenic share of C
Paper	36 %	85,00 %	43 %	0 %
		(Astrup et al., 2009)	(Nasrullah et al., 2014	(Larsen et al., 2013)
Plastic	24 %	91,50 %	74 %	100 %
(hard)		(Astrup et al., 2009)	(Nasrullah et al., 2014	(Larsen et al., 2013)
Plastic (soft)	17 %	91,50 %	75 %	100 %
		(Astrup et al., 2009)	(Nasrullah et al., 2014	(Larsen et al., 2013)
Textile	9 %	86,00 %	57 %	25 %
		(Astrup et al., 2009)	(Nasrullah et al., 2014	(Astrup et al., 2009)
Wood	6 %	80,00 %	49 %	0 %
		(Tchobanoglous et al., 1993)	(Nasrullah et al., 2014(Nasrullah et al., 2015)	(Larsen et al., 2013)
Rubber	1 %	98,00 %	48 %	0,00 %
		(Tchobanoglous et al., 1993)	(Nasrullah et al., 2014	(Kunioka et al., 2014)
Foam	1 %	NA	NA	NA
Metal	1 %	90,00 %	0,2 %	50 %
		(Astrup et al., 2009)	(Astrup et al., 2009)	(Astrup et al., 2009)
Glass	0 %	92,00 %	0,7 %	50 %
		(Astrup et al., 2009)	(Astrup et al., 2009)	(Astrup et al., 2009)
Stones	0 %	NA	NA	NA
Fines	6 %	NA	NA	NA

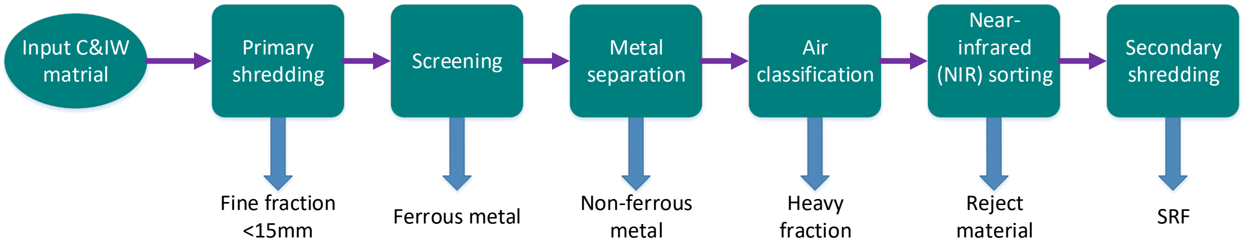

Figure 1. Mechanical treatment process of SRF production from C&IW material (Nasrullah et al., 2014).

Table 6. LHVar and CO2 emission factor of fuels (Khan, 2018)

Fuel types	LHV_{ar} (MJ/kg)	CEF_e (g CO_2, eq./MJ fuel)	CEF_m (g CO_2, eq./kg fuel)
Coal	25	95	2375
Petroleum coke	32	94	3008
Waste oil	30	77	2310
LFO	43	77	3311
SRF	20	50	1000

CEF_e = emission factor based on fuel energy, CEF_m emission factor based on fuel mass.

Table 7. Composition of C&IW and reject material (Nasrullah et al., 2014).

Components	C&IW (%)	Reject material (%)
Paper & cardboard	31	12
Plastic(soft)	17	14
Plastic(hard)	15	5
Textile	9	9
Wood	7	4
Rubber	3	7
Foam	1	0
Metal	6	3
Glass	4	10
Stones	3	19
Fines	5	17

Table 8. Total solid and non-biogenic carbon of C&IW components.

C&IW	Total solid content (TS)	Total share of carbon (C)	Non-biogenic share of C
Paper	85,00 % (Astrup et al., 2009)	43 % (Nasrullah et al., 2014	0 % (Larsen et al., 2013)
Plastic(hard)	91,50 % (Astrup et al., 2009)	74 % (Nasrullah et al., 2014)	100 % (Larsen et al., 2013)
Plastic(soft)	91,50 % (Astrup et al., 2009)	75 % (Nasrullah et al., 2014	100 % (Larsen et al., 2013)
Textile	86,00 % (Astrup et al., 2009)	57 % (Nasrullah et al., 2014	25 % (Astrup et al., 2009)

Wood	80,00 % (Tchobanoglous et al., 1993)	49 % (Nasrullah et al., 2014	0 % (Larsen et al., 2013)
Rubber	98,00 % (Tchobanoglous et al., 1993)	48 % (Nasrullah et al., 2014	0,00 % (Kunioka et al., 2014)
Foam	NA	NA	NA
Metal	90,00 % (Astrup et al., 2009)	0,2 % (Astrup et al., 2009)	50 % (Astrup et al., 2009)
Glass	92,00 % (Astrup et al., 2009)	0,7 % (Astrup et al., 2009)	50 % (Astrup et al., 2009)
Stones	NA	NA	NA
Fines	NA	NA	NA

Table 9. Total solid and non-biogenic carbon of reject material components.

Reject material	Total solid content (TS)	Total share of carbon (C)	Non-biogenic share of C
Paper	85,00 % (Astrup et al., 2009)	43 % (Nasrullah et al., 2014	0 % (Larsen et al., 2013)
Plastic(hard)	91,50 % (Astrup et al., 2009)	74 % (Nasrullah et al., 2014	100 % (Larsen et al., 2013)
Plastic(soft)	91,50 % (Astrup et al., 2009)	75 % (Nasrullah et al., 2014)	100 % (Larsen et al., 2013)
Textile	86,00 % (Astrup et al., 2009)	57 % (Nasrullah et al., 2014	25 % (Astrup et al., 2009)
Wood	80,00 % (Tchobanoglous et al., 1993)	49 % (Nasrullah et al., 2014)	0 % (Larsen et al., 2013)
Rubber	98,00 % (Tchobanoglous et al., 1993)	48 % (Nasrullah et al., 2014	0,00 % (Kunioka et al., 2014)
Foam	NA	NA	NA
Metal	90,00 % (Astrup et al., 2009)	0,2 % (Astrup et al., 2009)	50 % (Astrup et al., 2009)
Glass	92,00 % (Astrup et al., 2009)	0,7 % (Astrup et al., 2009)	50 % (Astrup et al., 2009)
Stones	NA	NA	NA

Fines	NA	NA	NA

Table 10. Relative contribution on GWP in cement production system.

	Thermal energy from SRF	SRF acquisition	Thermal energy from fossil fuels	Fossil fuels acquisition	Calcination	Electricity Consumption	Raw materials acquisition
SC 1	0 %	0 %	35 %	5 %	55 %	3 %	2 %
SC 2	0 %	0 %	35 %	5 %	55 %	3 %	2 %
SC 3	10 %	0 %	18 %	4 %	62 %	3 %	2 %
SC4	12 %	1 %	9 %	1 %	71 %	4 %	2 %

References

Astrup, T., Møller, J., Fruergaard, T., 2009. Incineration and co-combustion of waste: accounting of greenhouse gases and global warming contributions. Waste Manag. Res. Res. 27, 789–799. https://doi.org/10.1177/0734242X09343774

cBalance Solutions Hub |, 2013. Carbon Saving achieved by Recycling.

Khan, M.M.H., 2018. GWP IMPACT OF UTILIZING SRF IN CEMENT PLANTS : FINNSEMENTTI CASE STUDY. Lappeenranta University of Technology.

Kunioka, M., Taguchi, K., Ninomiya, F., Nakajima, M., Saito, A., Araki, S., Kunioka, M., Taguchi, K., Ninomiya, F., Nakajima, M., Saito, A., Araki, S., 2014. Biobased Contents of Natural Rubber Model Compound and Its Separated Constituents. Polymers (Basel). 6, 423–442. https://doi.org/10.3390/polym6020423

Larsen, A.W., Fuglsang, K., Pedersen, N.H., Fellner, J., Rechberger, H., Astrup, T., 2013.

Biogenic carbon in combustible waste: Waste composition, variability and measurement uncertainty. Waste Manag. Res. 31, 56–66. https://doi.org/10.1177/0734242X13502387

Nasrullah, M., Vainikka, P., Hannula, J., Hurme, M., Kärki, J., 2014. Mass, energy and material balances of SRF production process. Part 1: SRF produced from commercial and industrial waste. Waste Manag. 34, 1398–1407. https://doi.org/10.1016/J.WASMAN.2014.03.011

Nasrullah, M., Vainikka, P., Hannula, J., Hurme, M., Koskinen, J., 2015. Elemental balance of SRF production process: Solid recovered fuel produced from construction and demolition waste. Fuel 159, 280–288. https://doi.org/10.1016/j.fuel.2015.06.082

Tchobanoglous, G., Theisen, H., Vigil, S.A., 1993. Integrated solid waste management: engineering principles and management issues.

Thinkstep, 2018. thinkstep [WWW Document]. URL https://thinkstep.com/software/gabi-software/gabi-professional (accessed 12.20.18).
